# Two-dimensional Dirac plasmon-polaritons in graphene, 3D topological insulator and hybrid systems

**DOI:** 10.1038/s41377-022-01012-2

**Published:** 2022-10-27

**Authors:** Chihun In, Un Jeong Kim, Hyunyong Choi

**Affiliations:** 1 Department of Physics, Freie Universität Berlin, Berlin, 14195 Germany; 2 Department of Physical Chemistry, Fritz-Haber-Institute of the Max-Planck-Society, Berlin, 14195 Germany; 3 Department of Physics and Astronomy, Seoul National University, Seoul, 08826 Republic of Korea; 4 Institute of Applied Physics, Seoul National University, Seoul, 08826 Republic of Korea; 5 Advanced Sensor Laboratory, Samsung Advanced Institute of Technology, Suwon, Gyeonggi-do 16419 Republic of Korea

**Keywords:** Terahertz optics, Photonic devices, Sub-wavelength optics

## Abstract

Collective oscillations of massless particles in two-dimensional (2D) Dirac materials offer an innovative route toward implementing atomically thin devices based on low-energy quasiparticle interactions. Strong confinement of near-field distribution on the 2D surface is essential to demonstrate extraordinary optoelectronic functions, providing means to shape the spectral response at the mid-infrared (IR) wavelength. Although the dynamic polarization from the linear response theory has successfully accounted for a range of experimental observations, a unified perspective was still elusive, connecting the state-of-the-art developments based on the 2D Dirac plasmon-polaritons. Here, we review recent works on graphene and three-dimensional (3D) topological insulator (TI) plasmon-polariton, where the mid-IR and terahertz (THz) radiation experiences prominent confinement into a deep-subwavelength scale in a novel optoelectronic structure. After presenting general light-matter interactions between 2D Dirac plasmon and subwavelength quasiparticle excitations, we introduce various experimental techniques to couple the plasmon-polaritons with electromagnetic radiations. Electrical and optical controls over the plasmonic excitations reveal the hybridized plasmon modes in graphene and 3D TI, demonstrating an intense near-field interaction of 2D Dirac plasmon within the highly-compressed volume. These findings can further be applied to invent optoelectronic bio-molecular sensors, atomically thin photodetectors, and laser-driven light sources.

## Abstract (web summary)

Graphene and topological insulators are promising platforms for near-field nanotechnology on a subwavelength scale. Authors present the highly confined nature of Dirac plasmon-polaritons in graphene, topological insulator, and hybrid systems.

## Graphical abstract

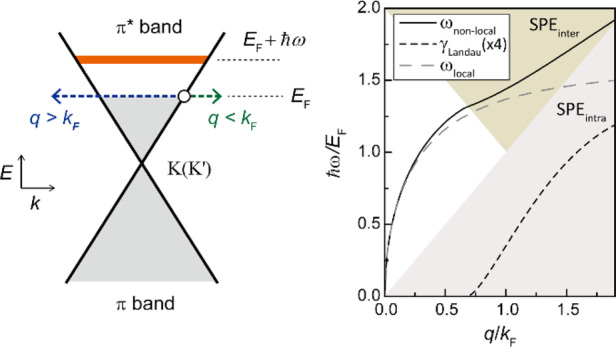

## Introduction

Graphene consists of hexagonal arrangements of carbon atoms in two-dimension (2D). The unit cell of graphene contains two equivalent carbon atoms, where the nearest-neighbor hopping interaction is based on the sp^2^ hybridization. Because the Bloch Hamiltonian in a unit cell is inversion symmetric, the gapless-linear band dispersion appears at the corner of the Brillouin zone, namely K and K′ points. Strikingly, the many-body interactions in graphene can be described based on the (2 + 1)-dimensional Dirac Hamiltonian. A range of optoelectronic measurements has demonstrated that graphene’s optical responses are different from those in the conventional two-dimensional semiconductor structures, primarily because of the 2D Dirac dispersion. The early studies on graphene focused on the transport measurements in a strong magnetic field^1,2^ verifying the half-integer quantum Hall (QH) conductivity *σ*_*xy*_ = (4*e*^2^/*h*)(*N* + 1/2), where *N* is integer. While the conventional 2D electron gas presents the standard integer QH state *σ*_*xy*_ ∝ (*e*^2^/*h*)*N*, the QH spectra in monolayer graphene are shifted by a half-integer, which can be elucidated by the (2 + 1)-dimensional Dirac Hamiltonian in a strong magnetic field. The gapless nature of graphene bandstructure also hosts the Dirac-fluid; the co-existence of electron and hole in the charge-neutral point has shown viscous charge flow^3–5^.

Plasmonics, the collective response of light-matter interaction, also shed light on the unique properties of graphene^6–9^. The light-induced particle-hole polarization of graphene can be calculated using random-phase approximation (RPA)^10–14^. The calculation results show that the graphene polarization is determined by two independent contributions of the intraband and interband polarization, where the Fermi energy distinguishes the two regimes. At the long-wavelength limit, the interband polarization promises a constant absorbance ~2.3% independent of light frequency^15^. In contrast, the intraband polarization explains the free carrier response against electromagnetic radiation^16–18^. Notably, light coupling with dipole momentum *q* exceeding photon *q*_*c*_ can excite the graphene plasmon-polariton (*q*/*q*_*c*_ ~ 10^2^). Here, the relationship of plasmon frequency *ω* versus the plsmon momentum *q* follows the conventional plasmon mode $\omega \propto \sqrt q$ based on noble 2D metals. However, the carrier-density (*n*) dependence allows us to distinguish the 2D Dirac plasmon (*ω* ∝ *n*^1/4^) from the conventional 2D plasmon (*ω* ∝ *n*^1/2^)^19^. A truly intriguing aspect of monolayer graphene is that the interparticle interaction is weak compared to conventional semiconductors, which is attributed to the linear dispersion of the gapless bandstructure^11^. The RPA result can precisely describe the 2D Dirac plasmon’s density-dependence in this weakly interacting system.

The near-field distribution arising from the charge oscillations in monolayer graphene can be coupled with novel optoelectronic structures^20–22^. Nonetheless, the scattering dynamics of Dirac fermions mainly limit the lifetime of 2D Dirac plasmon^23–25^. The momentum relaxation mechanism at low carrier density is primarily attributed to the impurity scatterings^24^, although the electron-phonon interaction still affects the carrier lifetime at high carrier density^25^. On the other hand, monolayer graphene encapsulated with hexagonal boron nitride (hBN) has shown high carrier mobility *μ* ~ 10^5^ cm^2^V^−1^ s^−1^ because of uniform interfaces of the van der Waals heterostructure^26^. With the low density of interface impurity, the hBN/graphene/hBN structures provide a sensitive tool to investigate the quasiparticle interactions. For instance, 2D Dirac plasmon can be coupled with phonon-polaritons^27–29^, where the plasmon energy is split into several branches near the phonon-polariton energy. The high mobility of monolayer graphene also enables us to detect an acoustic plasmon mode^30–34^, which strongly confines the plasmon momentum into a deep-subwavelength limit. Notably, the compressed graphene polarization unravels electron-electron interactions at low carrier density^31^.

For the ideal case of graphene, which assumes sp^2^ hybridization for the crystal bonding, the gapless-linear dispersion appears at the K and K′ points in the Brillouin zone. In reality, however, the spin–orbit coupling can open up the bandgap via the *σ*-π bond hybridizations^35–37^. The induced bandgap is a tiny perturbation ~μeV compared to the typical Fermi-energy scale observed in graphene; thereby, it is safe to use the ideal 2D Dirac dispersion to describe the light-matter interactions. Nonetheless, the bulk bandgap arising from the spin-orbit coupling produces highly non-trivial results at the graphene boundary: the bandgap is replaced by a spin-dependent one-dimensional (1D) conduction channel. A significant aspect of the 1D boundary state is that the spin direction is orthogonal to the direction of particle momentum. The spin-filtered transport promises dissipationless conduction as long as the spin-orbit coupling sustains the bulk bandgap in monolayer graphene. The possible existence of the non-trivial boundary state could be experimentally confirmed in other crystals with strong spin-orbit interaction^38,39^. Crucially, the boundary state of three-dimensional (3D) crystal can host 2D conduction at the surface, where the spin direction is orthogonal to surface normal vector and momentum direction. 3D topological insulators (TI), Bi_2_Se_3_, Bi_2_Te_3_, and Sb_2_Te_3_, are known to show the non-trivial boundary state on the surface^40,41^. Essentially, the surface state of 3D TI follows 2D Dirac dispersion similar to the case of graphene, except that the spin- and valley-degeneracy are absent on the 3D TI surface. Therefore, the 3D TI surface hosts 2D Dirac plasmon^42–44^, while the bulk inside TI spatially separates the opposite surface states, screening the near-field emanating from the plasmon-polariton.

This review provides a comprehensive analysis of 2D surface plasmon-polariton (SPP) in the gapless Dirac-band dispersions of graphene and 3D TI. The SPP in graphene and 3D TI uncover the unique light-matter interaction in the associated 2D Dirac-band. The electric field oscillations from SPP are confined up to a few atomic layers, which could amplify Coulomb interactions with neighboring materials. The following section addresses general aspects of 2D Dirac plasmon in terms of dispersion relations calculated from RPA. After presenting the dispersion relation in a monolayer 2D Dirac-band, we proceed with the interactive systems incorporating quasiparticle excitations. Furthermore, we introduce basic experimental techniques to couple electromagnetic waves with plasmon-polariton, presenting novel methods to control the 2D Dirac plasmon in a deep-subwavelength scale. The electrical and optical modulations are achieved in a graphene field-effect device and graphene-integrated 3D TI structure. As to provide future outlooks for the 2D Dirac plasmon, we list state-of-art applications of graphene plasmon at the end of this review.

## 2D Dirac plasmon-polariton dispersion

**Fig. 1:**
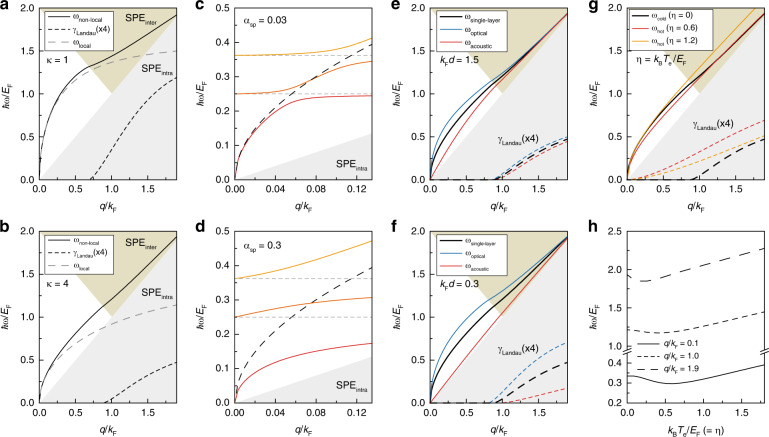
Two-dimensional (2D) Dirac plasmon-polariton dispersion. **a** Numerical calculation results are displayed, where the plasmon frequency *ω* and wavevector *q* are divided by Fermi energy *E*_F_ and Fermi wavevector *k*_F_, respectively. The *ω*_non-local_ (*ω*_local_) is calculated from the non-local (local) graphene polarization. The imaginary part of the plasmon mode (*γ*_Landau_) is obtained from the non-local graphene polarization, emergent when the *ω* enters either the interband (inter) or intraband (intra) single-particle-excitation (SPE) region. Note that the *γ*_Landau_ is multiplied by 4 for comparison. The background dielectric constant is *κ* = 1. **b** Plasmon modes are displayed with *κ* = 4. **c** Plasmon-phonon couplings split the unperturbed plasmon mode (black dashed line) into hybridized modes (colored solid lines). Horizontal dashed lines indicate surface phonon-polariton energies in silicon oxide. The plasmon-phonon coupling constant is *α*_sp_ = 0.03. **d** Equivalent results are obtained at *α*_sp_ = 0.3. **e** Double-layer graphene structure splits monolayer graphene plasmon (*ω*_single-layer_) into the optical (*ω*_optical_) and acoustic plasmon mode (*ω*_acoustic_). The *γ*_Landau_ in the monolayer graphene (black dashed line) is also split into optical (blue dashed line) and acoustic *γ*_Landau_ (red dashed line). The distance between the double layer is *k*_F_*d* = 1.5. **f** Equivalent calculations with *k*_F_*d* = 0.3 are shown. **g** Graphene plasmons at cold (*ω*_cold_, *η* = 0) and hot plasma cases (*ω*_hot_, *η* > 0) are displayed with *η* = *k*_B_*T*_e_/*E*_F_. The *γ*_Landau_ of *ω*_cold_ (black dashed line) is lower than *γ*_Landau_ at *η* = 0.6 (red dashed line) and *η* = 1.2 (yellow dashed line). **h** The *ω* as a function of *η* are plotted at different *q*

The energy and momentum dispersions of SPP characterize the longitudinal oscillations of free carriers on metallic films. The local approximation *q* → 0 of 2D Dirac plasmon dispersion follows the sublinear relationship $\omega \propto \sqrt q$ similar to the conventional 2D electron gases. At a deep-subwavelength regime, however, the non-local response of 2D Dirac plasmon deviates^31^ from the conventional SPP dispersion. When *q* is nested at the Fermi surface, additional photon energy is required to excite SPP above the Fermi energy, which results in the blue-shift in the SPP dispersion. Recent studies on graphene highlight the significance of non-local response resulting from the strongly-confined plasmon wavelength (*λ*_p_)^31^. Figure 1 depicts the SPP dispersion of 2D Dirac-bands, where the plasmon frequency *ω* and wavevector *q* are normalized by the Fermi energy *E*_F_ and Fermi wavevector *k*_F_, respectively. Here, we employed the full description of the non-local graphene polarization P(*q*,*ω*) derived from the RPA (Eq. (9) in ref. ^10^), finding zeros of the dielectric function *ε*(*q,ω*) = 1−*υ*_q_P(*q,ω*), where *υ*_q_ = *e*^2^/2*κ*_0_*κq* is the Fourier-transformed 2D Coulomb interaction. Figure 1a, b compare the SPP dispersion when the background dielectric constant *κ* changes, where the screening effect of the surrounding medium shifts the plasmon dispersion toward the lower frequency regime. Although the local approximation *ω*_local_ obtained from P_local_(*q,ω*) (Eq. (12) in ref. ^10^) follows the non-local SPP (*ω*_non–local_) at the long wavelength limit (*q* → 0), it deviates when the *q* is comparable to *k*_F_, implying that the SPP is nested at the Fermi surface. SPP can excite interband particle-hole pairs when entering the single-particle excitation (SPE) region. The intrinsic SPP decay rate is characterized by the Landau damping *γ*_Landau_, which becomes significant at the deep-subwavelength regime. The rest this section embraces the non-local P(*q,ω*) to calculate the SPP dispersion with an average dielectric constant *κ* = 4 of the surrounding medium. This is because graphene is surrounded by dielectric materials in general. Besides, the RPA calculation is appropriate for weakly interacting particles, which requires the screening effect of the surrounding medium. The Wigner-Seitz radius *r*_*s*_ is a brief measure of the interparticle interactions *r*_*s*_ = (*α*/*κ*)(*c*/*v*_F_)(4/*g*_*s*_*g*_*v*_)^1/2^, where *r*_*s*_ ≪ 1 implies weak particle interactions^11^. The screening factor *κ* = 4 lowers the *r*_*s*_ value to ~0.5, where we assume the fine-structure constant *α* = 1/137, the graphene Fermi velocity *v*_F_ = 1 × 10^6^ m/s, and spin- and valley-degeneracies *g*_*s*_*g*_*v*_ = 4.

Charge oscillations in an atomically thin layer experience strong Coulomb interactions when the dielectric constant rapidly changes as a function of frequency. This effect is pronounced near the surface optical (SO) phonon frequencies (*ω*_SO_)^45–47^, where the SPP dispersion is split into upper and lower branches at the vicinity of *ω*_SO_. The energy splitting is characterized by the coupling constant *α*_sp_ = *ε*_∞_[(*ε*_∞_ + 1)^−1^−(*ε*_0_ + 1)^−1^], where *ε*_∞_ (*ε*_0_) is the high-frequency (static) dielectric constant. Figure 1c and d display the SPP dispersion modified by the plasmon-phonon coupling, finding zeros of the dielectric function^13^

1 $$\varepsilon \left( {q,\omega } \right) = 1 - \upsilon _{{{\mathrm{q}}}}{{{\mathrm{P}}}}\left( {q,\omega } \right) - \left[ {1 + \left[ {e^{ - 2qd}\left( {\frac{{\omega _{{{{\mathrm{SO}}}}}}}{2}} \right)\alpha _{{{{\mathrm{sp}}}}}{\sum} {D_0\left( \omega \right)} } \right]^{ - 1}} \right]^{ - 1}$$

where $D_0\left( \omega \right) = 2\omega _{{{{\mathrm{SO}}}}}/(\omega ^2 - \omega _{{{{\mathrm{SO}}}}}^2)$ is the SO-phonon propagator and *d* = 0.5 nm is the distance between the graphene and substrate. Here, we assumed the graphene *E*_F_ = 0.4 eV above the silicon oxide SO-phonon energies of 100 meV and 145 meV. Clearly, *α*_sp_ increases the energy splitting in the SPP dispersion. The lowest branch of the phonon-coupled SPP is damped upon entering the SPE_intra_ region, which requires extreme confinement of *λ*_p_ and the large plasmon-phonon coupling. Yan et al.^45^ reported that the graphene optical phonon (~0.2 eV) assists the plasmon damping into intraband particle-hole pairs, where the inelastic damping manifests as the broadened SPP resonance. On the other hand, the hyperbolic phonon-polariton (HPP) can propagate inside a dielectric material with anisotropic phonon-polariton resonances^48,49^. Thin films of hBN host the HPP energy bands at 90–100 meV (type I) and 175–200 meV (type II). The plasmon-phonon coupling inside the HPP bands is different from the SO phonon-plasmon coupling because the HPP mode also disperses with the polariton wavevector. These considerations are discussed elsewhere^27,50^.

The potential application of the graphene plasmon includes an atomically thin photodetector, where the multiple stacks of monolayer graphene can amplify the photoresponsivity^51,52^. When the distance of the multiple stacks is comparable to the plasmon wavelength, the interlayer Coulomb interaction splits the SPP into the optical plasmon and acoustic plasmon modes. Figure 1e, f display the split of the SPP dispersion in double-layer graphene, where we assumed the same Fermi energy of top (T) and bottom (B) layers to address the effect of the interlayer Coulomb interaction. We find the zeros of the dielectric function of the double-layer graphene^12,53^ separated by the distance *d*,

2 $$\begin{array}{l}\varepsilon \left( {q,\omega } \right) = \left[ {1 - \upsilon _{{{\mathrm{q}}}}{{{\mathrm{P}}}}_{{{\mathrm{T}}}}\left( {q,\omega } \right)} \right]\left[ {1 - \upsilon _{{{\mathrm{q}}}}{{{\mathrm{P}}}}_{{{\mathrm{B}}}}\left( {q,\omega } \right)} \right]\\\qquad\qquad\; -\, \upsilon _{{{\mathrm{q}}}}^2e^{ - 2qd}{{{\mathrm{P}}}}_{{{\mathrm{T}}}}\left( {q,\omega } \right){{{\mathrm{P}}}}_{{{\mathrm{B}}}}\left( {q,\omega } \right)\end{array}$$

The decrease in *d* increases the Coulomb interaction, increasing the split energy between the optical and acoustic modes. The dynamic charge distribution of the double-layer plasmon is symmetric (anti-symmetric) along with the normal axis of the optical (acoustic) plasmon mode. At the long-wavelength limit *q* → 0, the dispersion of the optical mode $\left( {\omega \propto \sqrt q } \right)$ distinguishes itself from the acoustic mode (*ω* ∝ *q*). Besides, the distinct charge distribution imposes a potential limit to the light coupling method. The far-field radiation mainly excites the optical plasmon mode since the distance of the double-layer graphene is generally far shorter than the photon wavelength in free space^52,54^. Recently, the acoustic plasmon mode in graphene has been demonstrated in a novel dual-gate structure^30,31^, where the asymmetric charge distribution is driven by the image charge reflected on the metallic gate side. As indicated in Fig. 1f, the phase velocity of the acoustic mode is suppressed right up to the Fermi velocity when *d* decreases. Strikingly, the decrease in *d* also curbs the acoustic mode’s damping rate, presumably because the out-of-plane dipole moment becomes significant in close proximity. On the other hand, the SPP response in 3D TI is analogous to the case of the double-layer graphene because the 3D TI surface states always come in a pair, where the TI bulk spatially separates the surface states. Notably, a general expression of dynamic screening function for the double-layer structure can be found in ref. ^53^, where the *ε*(*q,ω*) includes different values of *κ* inside and outside of the 2D Dirac layers.

The remainder of this section is devoted to addressing the intrinsic 2D Dirac plasmon dispersion under the hot Fermi-Dirac distribution $f\left( {E,E_{{{\mathrm{F}}}}} \right) = \left[ {e^{\left( {E - E_{{{\mathrm{F}}}}} \right)/k_{{{\mathrm{B}}}}T} + 1} \right]^{ - 1}$. At room temperature and a moderate doping level ~100 meV, the thermal excitation makes a minimal change in the SPP dispersion. However, in some experiments, the high laser pulse intensity can elevate the electron temperature *T*_e_ above 1000 K, creating the hot Dirac plasma^55–57^. Figure 1g shows how the 2D Dirac plasmon dispersion evolves when the thermal energy *k*_B_*T*_e_ exceeds *E*_F_, where we introduce a characteristic constant *η* = *k*_B_*T*_e_/*E*_F_ to compare the intrinsic plasmon to the cold plasma case (*η* = 0). The intrinsic plasmon dispersion can be obtained from the zeros of the dielectric function 1 − *υ*_q_P(*q, ω, μ*; *T*_e_) = 0 with the polarization^14^

3 $${\rm{P}}\left( {q,\omega ,\mu ;{T_{\rm{e}}}} \right) = \int_0^\infty {d{\mu ^\prime}\frac{{{\rm{P}}\left( {q,\omega ,{\mu ^\prime};{T_{\rm{e}}} = 0} \right)}}{{4{k_{\rm{B}}}{T_{\rm{e}}}{{\cosh }^2}\left[ {\left( {\mu - {\mu ^\prime}} \right)/2{k_{\rm{B}}}{T_{\rm{e}}}} \right]}}}$$

where *μ*(*T*_e_) is the chemical potential following the charge conservation $n = {\int}_0^\infty {D\left( E \right)\left[ {f\left( {E,\mu } \right) - f\left( {E, - \mu } \right)} \right]dE}$ with the 2D Dirac density-of-state *D*(*E*) = *g*_*s*_*g*_*v*_*E*/2πℏ^2^*v*_F_^2^. When *η* > 1, the interband thermal excitation increases the sum of the electron and hole density following $n_T \propto T_{{{\mathrm{e}}}}^2$. Recalling that the SPP dispersion shows the sublinear dependence on the carrier density *ω* ∝ *n*^1/4^, it is evident that the increase in *ω* arises from the thermal population of the 2D Dirac bands^14^. However, at the intermediate state (*η* = 0.6), *ω* is somewhat decreased compared to the cold plasma case, implying that the 2D Dirac plasmon non-monotonically depends on the temperature. Unlike the conventional 2D electron gas, *D*(*E*) in 2D Dirac-bands is proportional to *E*. As a result, the intraband thermal excitation always accompanies the decrease in *μ*(*T*_e_), which explains the red-shift in *ω* at the intermediate state. Figure 1h shows the initial decrease and the subsequent increase of *ω* as a function of *η*. Meanwhile, the SPP encounters Landau damping *γ*_Landau_ before entering the SPE region because of the free carriers smeared from the Fermi surface (Fig. 1g).

## Excitations of 2D Dirac plasmon-polariton

**Fig. 2:**
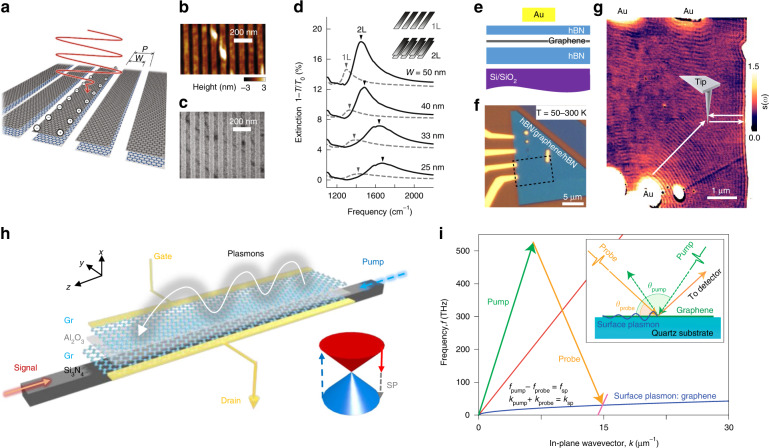
Excitations of 2D Dirac plasmon-polaritons. **a** Schematic illustration of double-layer graphene nanoribbon indicates period *P* and width *W*. **b** Surface topography image measured by atomic force microscopy and **c** scanning electron microscopy. **d** Spectral extinction 1−*T*/*T*_0_ at monolayer (1 L) and double-layer graphene nanoribbon (2 L) at different *W*. **e**, **f** Schematic illustration of (**e**) Au/hBN/graphene/hBN/SiO_2_/Si device and (**f**) its optical microscopic image. **g** s-SNOM image is shown with the SPP propagation direction (white arrows). **h** Double-layer graphene Gr/Al_2_O_3_/Gr is transferred onto Si_3_N_4_ waveguide where the pump (dashed blue line) is converted into signal (solid red line) and surface plasmon SP (gray line). The gate voltage simultaneously changes the doping level of the top and bottom Gr. **i** Difference-frequency generation of graphene surface plasmon (solid blue line) with pump (green arrow) and probe (orange arrow). The red line indicates light dispersion inside quartz substrate. Inset: pump and probe beams are incident on graphene on quartz substrate with angle *θ*_pump_ and *θ*_probe_, respectively. **a**–**d** Adapted with permission from ref. ^54^, Springer Nature. **e‒g** Adapted with permission from ref. ^65^, Springer Nature. **h** Adapted with permission from ref. ^69^, Springer Nature. **i** Adapted with permission from ref. ^68^, Springer Nature

This part introduces experimental techniques to excite plasmon-polariton in graphene and 3D TI. In general, the plasmon wavelength *λ*_p_ is far shorter than the photon wavelength in the vacuum *λ*_0_ by two orders of magnitude (*λ*_p_/*λ*_0_ = 10^2^), where the strong confinement is ascribed to the slow Fermi velocity of graphene (*v*_F_/*c* = 1/300). One intuitive way to acquire an additional momentum *q* = 2*π*/*λ*_p_ is to fabricate a nanoribbon structure^43,45,58^, where the period *P* gives rise to the plasmon momentum *q* = 2*π*/*P*. Figure 2a–d show that the graphene nanoribbon structure can be used to excite a monolayer and double-layer 2D Dirac plasmon^54^. The electric field perpendicular to the nanoribbon confines the polarization in the nanoribbon width *W* upon the laser irradiation. As shown in Fig. 2d, the graphene SPP leads to the resonant extinction spectra, where the resonance center and the resonance width define the plasmon frequency and damping, respectively. As mentioned earlier, the decrease in *W* (increase in *q*) increases the plasmon frequency, following the sublinear dispersion of the 2D Dirac plasmon $\omega \propto \sqrt q$. The blue shift in the double-layer graphene indicates that the incident light excites the optical plasmon mode, where spectral extinction is stronger than the monolayer case.

On the other hand, the scattering-type scanning near-field microscopy (s-SNOM) allows us to acquire the near-field profiles of the graphene plasmon^59,60^ and TI plasmon^61,62^, tailoring the phase and propagation of the excited SPP^63,64^. Figure 2e, f display the graphene field-effect device capped with hBN, where the near-field images reproduce the device structure. Here, an atomic force microscope (AFM)-tip is irradiated by a focused laser beam (*λ*_0_ = 11.28 μm), confining the photon wavelength into the deep subwavelength scale. The SPP launched by the AFM-probe spreads out away from the source, which experiences reflection at the edge of the graphene flake. A prominent light scattering occurs when the reflected SPP reaches the AFM-probe again, producing the near-field modulations depending on the AFM-tip position (Fig. 2g). Sweeping the AFM-probe on the graphene flake, Ni et al.^65^ was able to acquire a deep subwavelength spatial resolution of ~10 nm, where a proper demodulation technique was used to isolate the SPP-induced scatterings from the other light scattering sources. Notably, the period of near-field fringes corresponds to either *λ*_p_/2 or *λ*_p_ depending on the position of the AFM-probe. The period *λ*_p_/2 is made when the plasmon travels twice the distance between the tip and edge of the graphene flake, while the period *λ*_p_ presents the plasmon directly launched at the Au emitter which propagates toward the tip. With a constant light frequency, the s-SNOM experiment captures the density-dependence of 2D Dirac plasmon $\left( {\lambda _{{{\mathrm{p}}}} \propto \sqrt n } \right)$^27,28^, comparing the fringe period as a function of graphene carrier density *n*.

Understanding the dissipation pathway of graphene plasmon is essential to develop graphene-based optoelectronic applications. In general, the scattering event of graphene SPP involves a range of sources: electron–electron scatterings^23^, electron–impurity scatterings^24^, electron–phonon scatterings^25^, and dielectric losses of environment materials, imposing an intrinsic limit to the SPP lifetime. The highest *Q*_p_ value of 130 is reported in the graphene plasmon using the s-SNOM measurement^65^, circumventing various scattering sources by lowering the temperature (~50 K) and encapsulating the graphene with hBN flakes. At the low temperature, the electron-phonon scattering is largely reduced, remaining the dielectric loss as a residual scatterer against the graphene SPP. As reported in ref. ^28^, capping the graphene with hBN plays a vital role in reducing the impurity scattering, where the *Q*_p_ can be increased by five times when transferring the graphene from Si/SiO_2_ (*Q*_p_ ~ 5) to hBN substrate (*Q*_p_ ~ 25). Besides, the impurity scattering can be reduced by increasing the free carrier density of graphene. On the other hand, the fabrication of graphene nanostructure may contribute to the plasmon damping, which takes place at the edge of graphene nanoribbon via the elastic processes^45^. Similar effects are extensively studied in metallic nanoparticles^66^.

Counter-propagating electromagnetic waves inside a nonlinear medium can generate a difference-frequency of the input frequencies. Such a nonlinear process can be used to excite the SPP in graphene^67–69^. Figure 2h, i show that the graphene SPP is driven by either the optical waveguide or free-space optical pulses. The opposite direction of pump and signal (probe) ensures that the photon energy (*f*_pump_−*f*_probe_ = *f*_SPP_) and momentum (*k*_pump_ + *k*_probe_ = *k*_SPP_) are conserved during the difference frequency generation (DFG). Here, the transverse magnetic (TM) polarizations of the pump and probe allow the efficient coupling of the evanescent electric field and the graphene SPP at the interface. The excitation of graphene SPP manifests in the transient increase of the probe-intensity since the pump-photon is converted to the probe-photon through the DFG process. Here, the extracted *f*_SPP_ and *k*_SPP_ from the transient change in the probe spectra follow the 2D Dirac plasmon dispersion. Proper tuning of photon energies yields the nonlinear conversion efficiency of 6 × 10^−5^ W^−1^ in the optical waveguide^69^ (Fig. 2h) and 6 × 10^−6^ W^−1^ in the free space (Fig. 2i)^68^. The spectral width of transient response can define the damping rate of the graphene SPP in the nonlinear process. Yao et al.^69^ reported that the optical waveguide could yield a high-quality factor (*Q*_p_) of ~50, greater than the value in graphene nanoribbons (*Q*_p_ ~ 5).

## Thermoelectric detection of graphene plasmon-polariton

**Fig. 3:**
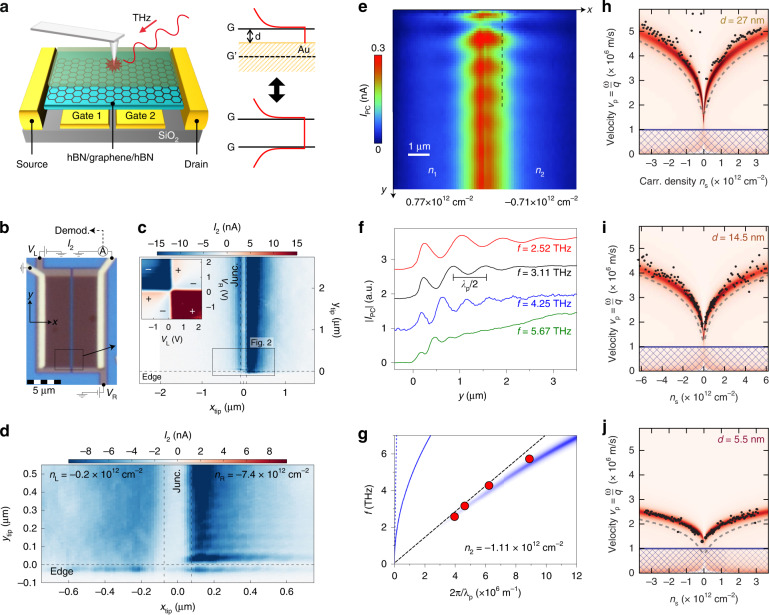
Conversion of graphene plasmon-polariton into thermoelectric photocurrent. **a** Schematic illustration of hBN/graphene/hBN transferred onto the SiO_2_/Si dual-gate field-effect device. The AFM-tip launches the SPP on the device. The distance between graphene (G) and Au is *d*, where the charge distribution is equivalent to the double-layer G. **b** Optical microscopic image of the dual-gate device, where the gate voltages *V*_L_ and *V*_R_ changes the Fermi energy. The demodulated signal *I*_2_ is measured at the right side of the electrode. **c** Photocurrent microscopy image of the dual-gate device. Inset: six-fold changes in *I*_2_ sign are shown at different *V*_L_ and *V*_R_. **d** An enlarged image of the box in (**c**) with carrier densities *n*_L_ = −0.2 × 10^12^ cm^−2^ (left) and *n*_R_ = −7.4 × 10^12^ cm^−2^ (right). **e** Photocurrent *I*_PC_ microscope image at different carrier densities on the left (*n*_1_ = 0.77 × 10^12^ cm^−2^) and right side (*n*_2_ = −0.71 × 10^12^ cm^−2^) of the dual-gate device. **f** Photocurrent fringes are measured along with the vertical dashed line indicated in (**e**). The frequency *f* is shifted from 2.52 THz to 5.67 THz. One period of the fringe corresponds to *λ*_p_/2. **g** Experimental results (red dots) at *n*_2_ = −1.11 × 10^12^ cm^−2^ are shown with the acoustic plasmon dispersion (blue color curve) and its first-order expansion at *λ*_p_ → ∞ (black dashed line). The plasmon dispersion of monolayer graphene (solid blue line) is displayed with the photon dispersion (blue dashed line). **h‒j** The plasmon phase velocity *v*_p_ (black dots) is obtained with *d* = 27 nm (**h**), *d* = 14.5 nm (**i**), and *d* = 5.5 nm (**j**) as a function of the carrier-density (*n*_*s*_). The red color maps are obtained from the non-local RPA with electron-electron interactions. The local approximations (dashed lines) are deviated from the measured data. **a**‒**d** Adapted with permission from ref. ^21^, Springer Nature. **e**‒**g** Adapted with permission from ref. ^30^, Springer Nature. **h**‒**j** Adapted with permission from ref. ^31^, American Association for the Advancement of Science

Photoresponse in doped graphene is based on thermoelectric^70^, photovoltaic^71^, and bolometric effects^72^. Whether the plasmonic excitation affects the photoresponse of graphene is of particular interest in the field of graphene-based optoelectronic applications^73^. Lundeberg et al.^21^ reported that the 2D Dirac plasmon can be employed as a thermoelectric photodetector in a dual-gated graphene field-effect device. Figure 3a–d show that the thermoelectric effect converts infrared light (*λ*_0_ = 10.6 μm) into the photocurrent *I*_2_. Here, the AFM-tip position (*x*_tip_ and *y*_tip_) modulates the *I*_2_ amplitude, indicating that the plasmonic excitation affects the graphene photoresponse. In fact, the AFM-tip position changes the absorption cross-section of the device; the light absorption can be enhanced by the constructive interference between the AFM-tip and the SPP reflected from the graphene edge, otherwise destructive interference reduces *I*_2_. The sixfold change in the *I*_2_ sign indicates that the thermoelectric effect is responsible for the photoresponse in the dual-gate device (inset in Fig. 3c). It is instructive to note that the thermoelectric voltage *V*_2_ is written as *V*_2_ = (*S*_*R*_ − *S*_*L*_)∆*T*, where *S*_*R*(*L*)_ is the Seebeck coefficient of the right (left) junction, and ∆*T* is the light-induced increase in the junction temperature. The gate voltage independently controls the carrier density of the right and left side of the junction. A closer look into the photocurrent map reveals that the *I*_2_ signal decays away from the junction with a decay length of ~250 nm (*x*_tip_-axis in Fig. 3d). The amplitude of *I*_2_ is pronounced at the high carrier density region since the coupling efficiency of the AFM-tip is higher at the longer plasmon wavelength *λ*_p_. On the other hand, the fringes along with the *y*_tip_-axis determine the plasmonic decay length, where the fringe period corresponds to *λ*_p_/2 similar to the case in the s-SNOM technique^65^. Significantly, the plasmon decay length (*y*_tip_-axis) was less than the *I*_2_ decay length (*x*_tip_-axis) at all carrier densities, indicating that the thermoelectric action is driven by the thermal diffusion rather than the direct transport of SPP into the junction.

The dispersion relation of 2D Dirac plasmon can be altered by surrounding electrostatic properties. In particular, an image charge of the graphene SPP can be reflected on a neighboring metallic film. As displayed in Fig. 3a, the graphene floating on top of gold is electrically equivalent to the double-layer graphene, where the signs of dynamic charges are opposite along the vertical axis^30^. For this reason, the dual-gate graphene field-effect device can launch the acoustic plasmon mode, given that the graphene-metal distance *d* is minimal compared to *λ*_p_. The frequency dispersion extracted from the near-field modulations can verify the acoustic plasmon mode. Figure 3e–g show the photoresponse in the dual-gate device, where the SPP excitation manifests in the fringes of thermoelectric photocurrent similar to the case in Fig. 3d. The incident light frequency lies in the terahertz (THz) wavelength (*λ*_0_ ~ 100 μm), which far exceeds the distance *d* = 42 nm of the device. As seen from Fig. 3f, the deep-subwavelength confinement produces near-field fringes of the THz wave, achieving a spatial resolution of ~50 nm. The light coupling at THz frequency can avoid the HPP excitation inside the hBN layers, otherwise hybridized dispersions might complicate identifying the acoustic plasmon mode. Besides, the THz radiation suppresses the interband transitions in graphene at low carrier density. Figure 3g shows that the SPP dispersion (red dots) launched in the dual-gate device deviates from the plasmon dispersion of monolayer graphene (solid blue line). Instead, the acoustic plasmon dispersion (blue color curve) correctly describes the highly-compressed status of the plasmon wavelength (*λ*_p_/*λ*_0_ ~ 1/66) in the dual-gate structure. Previously, we have addressed that the phase velocity *v*_p_ = *ω*/*q* of the acoustic plasmon mode approaches the Fermi velocity (*v*_F_ = 10^6^ m/s in graphene) when *d* is decreased. As demonstrated in ref. ^31^, *v*_p_ are suppressed up to *v*_F_ when the *d* decreases from 27 nm to 5.5 nm in the dual-gate device (Fig. 3h–j). The density-dependence of *v*_p_ (color maps) deviates from the long-wavelength limit (dashed lines), explaining the non-local response at the low carrier density.

## Intrinsic plasmon in graphene and 3D topological insulator

**Fig. 4:**
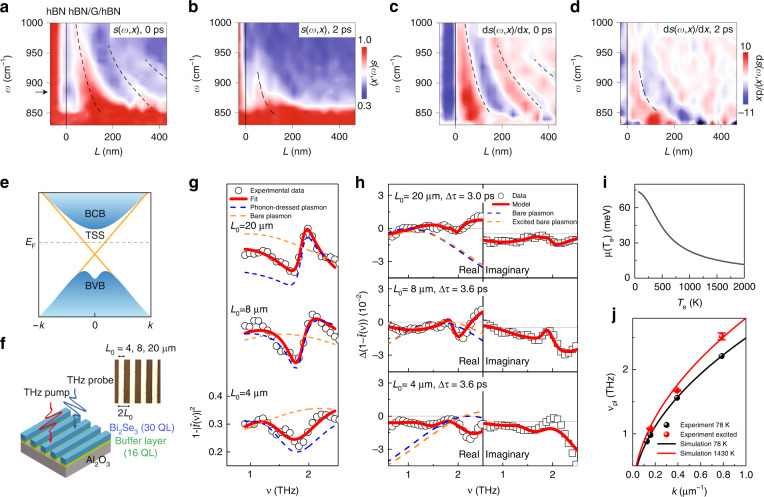
Intrinsic plasmons in graphene and 3D topological insulator. **a** s-SNOM signals *s*(*ω,x*) at 0 ps are shown as a function of distance *L* from the hBN/G/hBN boundary. **b** *s*(*ω,x*) at 2 ps. **c**, **d** The spatial derivatives d*s*(*ω,x***)**/d*x* are shown at 0 ps (**c**) and 2 ps (**d**). **e** Schematic illustration of Bi_2_Se_3_ bandstructure with topological surface state (TSS), bulk conduction band (BCB), and bulk valence band (BVB). **f** 30 QL Bi_2_Se_3_ microribbons is grown on (Bi_0.5_In_0.5_)_2_Se_3_/Al_2_O_3_. The electric-field polarization of the THz-pump (THz-probe) is parallel (perpendicular) to the microribbon’s direction. The width of the microribbon *L*_0_ is 4, 8, and 20 μm. **g** Spectral extinction (circles) are shown with fit lines (solid red line) at different *L*_0_. The fit lines are composed of the phonon-dressed plasmon (blue dashed line) and the bare plasmon extinction (orange dashed line). **h** Pump-induced change in the extinction (circles) are shown with fit lines (solid red line) at different *L*_0_. The plasmon extinction shifts from equilibrium (blue dashed line) to non-equilibrium (orange dashed line). The imaginary part (right column) is obtained from Kramers-Kronig relation of the real part (left column). **i** The TSS chemical potential *μ*(*T*_e_) is plotted as a function of the electron temperature *T*_e_. **j** The Bi_2_Se_3_ plasmon frequency *ν*_pl_ at *T*_e_ = 78 K (black dots) and *T*_e_ = 1430 K (red dots) are displayed as a function of the plasmon wavevector *k* = *π*/*L*_0_. The simulations (black and red lines) are performed at corresponding *T*_e_. **a**‒**d** Adapted with permission from ref. ^55^, Springer Nature. **e**‒**j** Adapted with permission from ref. ^57^, American Physical Society

So far, we have addressed that an exotic geometry of graphene field-effect device is able to modify the 2D Dirac plasmon dispersion, transferring the SPP into the deep-subwavelength scale. Likewise, the non-local response may emerge when optical pulses drive interband thermal excitations. Ni et al.^55^ reported that the electron temperature of graphene reaches 3200 K when using the ultrashort pulses (40 fs) of the near-infrared (IR) radiation (*λ* = 1.56 μm). In Fig. 4a–d, the s-SNOM measurement records the transient increase of *λ*_p_ induced by the near-IR pump, where the mid-IR pulses (~200 fs) are resonantly coupled with the AFM-tip, leaving the near-field fringes as a function of the tip position. As displayed in Fig. 4a, the pump-induced scattering signal *s*(*ω,x*) is pronounced at the temporal overlap of near-IR pump and mid-IR probe pulses, i.e. at the zero pump-probe delay. The fringe intervals of *s*(*ω,x*) (black dashed lines) reveal that the pump-induced *λ*_p_ is inversely proportional to the mid-IR frequency *ω*, which characterizes the 2D Dirac plasmon dispersion under the optical excitations. Notably, the derivatives of scattering signal (d*s*/d*x*) shown in Fig. 4c confirm the spectral and spatial peaks of *s*(*ω,x*). The pump-induced signal diminishes at the probe delay of 2 ps (Fig. 4b, d), following the conventional relaxation dynamics of hot carriers in graphene.

Having identified the thermal effect on the graphene plasmon, we now present the 2D Dirac plasmon on the topological insulator (TI) surface, where an intense terahertz (THz) radiation drives the non-equilibrium plasmonic dynamics^57^. As we discussed earlier, the bandstructure of 3D TI consists of an insulator-like bulk band and semi-metallic topological surface state (TSS). The large bandgap in Bi_2_Se_3_ (~0.3 eV) allows us to access the TSS response, hosting the 2D Dirac plasmon at the THz band. In Fig. 4e, the Bi_2_Se_3_ bandstructure is projected onto the Γ point of the surface Brillouin zone, where the TSS Fermi velocity is ~6 × 10^5^ m/s according to the angle-resolved photoemission measurement^74^. The molecular-beam-epitaxy (MBE) synthesizes 30 quintuple layers (QLs, 1 QL = 1 nm) of Bi_2_Se_3_ on (Bi_0.5_In_0.5_)_2_Se_3_ buffer layer, preventing the impurities arising from the lattice mismatch^75^. In general, pristine 3D TI films are degenerately doped because of vacancy-defect sites at the surface and crystalline defects in the bulk. With the decrease in the defects via interface engineering, a truly bulk-insulating status of 3D TI is obtained. The microribbons of Bi_2_Se_3_ provide the plasmon momentum *k* = *π*/*L*_0_, which is coupled with the THz-probe electric-field perpendicular to the ribbon direction. On the other hand, the THz-pump with an electric-field intensity ~ 0.4 MV/cm is incident parallel to the ribbon direction (Fig. 4f). The THz extinction measurement in Fig. 4g shows that the spectral response without the THz-pump undergoes blue-shift when *L*_0_ decreases. Here the Bi_2_Se_3_ $E_u^1$-phonon resonance at ~2 THz modifies the plasmon spectra, leaving an asymmetric profile at the phonon frequency. Lee et al.^57^ has extracted the bare plasmon extinction (orange dashed line) from the phonon-dressed plasmon spectra (solid red line) by employing the conventional plasmon-phonon coupling model^76,77^.

When the photon energy is larger than the Bi_2_Se_3_ bulk bandgap, the optical response in TSS is entangled with the bulk state. The low photon energy of the THz-pump (~4 meV), on the other hand, can avoid the unwanted carrier excitation in bulk, achieving the thermal distribution of the TSS with *T*_e_ above ~1400 K. As we have shown in Fig. 1h, the thermal energy *k*_B_*T*_e_ exceeding the Fermi energy *E*_F_ increases the plasmon frequency *ν*_pl_ due to the interband thermal excitations. Hence, the small value of Bi_2_Se_3_ *E*_F_ ~ 75 meV allows the interband thermal excitation when *k*_B_*T*_e_ ~ 120 meV is provided. Figure 4h shows that the THz-pump changes the extinction response, where the fit curves (solid red lines) is used to extract the spectral blue-shift of the TSS plasmon (blue and orange dashed lines). The imaginary part of the fit curve is obtained from the Kramer-Kronig relations of the real part. Comparing the TSS plasmons with and without the THz-pump, we identify that the amount of the blue-shift increases when the *L*_0_ value decreases, similar to the result observed in graphene (Fig. 4a). Figure 4i shows that the TSS chemical potential *μ*(*T*_e_) decreases when *T*_e_ increases, following the charge conservation law. Nonetheless, the strong interband thermal excitation increases *ν*_pl_ at all *k* values, demonstrating the intrinsic TSS plasmon under the THz excitations (Fig. 4j).

As we have discussed in this section, irradiation of laser pulses can drive the ultrafast dynamics of 2D Dirac plasmons, where the transient increase of *T*_e_ is responsible for the change in the plasmon dispersion. The electronic thermal energy relaxes through the electron-phonon interaction, transferring the excess energy to the thermal bath within a few picoseconds^78^. Pulsed radiation of THz wave is a reliable tool to track the ultrafast dynamics of 2D Dirac plasmon, where the Fourier-transformed analysis allows us to obtain the change in the spectral response. In Bi_2_Se_3_ TIs, the photon energy above the bulk bandgap (~0.3 eV) can trigger photoexcitation inside bulk, increasing the free carrier density transiently. According to the time-resolved THz spectroscopy^79–82^ and angle-resolved photoemission spectroscopy^83^, the recombination of the photoinduced bulk carriers shows a similar timescale to the cooling dynamics of thermal energy. For this reason, THz-pump as a driving source is indispensable for measuring the intrinsic plasmon dynamics in Bi_2_Se_3_, because the low photon energy ~4 meV does not contribute to the interband photoexcitation. In contrast, when the 1.55 eV optical-pump drives the bulk photoexcitation, the time-resolved THz spectroscopy reveals a large blue-shift in the plasmon frequency^77,84^, revealing the density-dependence of the conventional 2D plasmon.

It is instructive to note that the nonlinear kinetics of TSS under the intense THz radiation is of general interest in the field of Dirac materials. Indeed, Luo et al.^81^ showed that the TSS scattering dynamics are different from the bulk scatterings under the THz-pump excitation, where the pump-induced TSS scattering suppresses the THz conductivity. On the other hand, a resonant coupling of THz-pump and the Bi_2_Se_3_ phonon at ~2 THz displaces the lattice ions from the equilibrium^85^, leaving coherent oscillations in the second-harmonic-generation of probe pulses. Recently, Schmid et al.^86^ observed the high-harmonic (HH) generation of intense THz photon below the Bi_2_Te_3_ bulk bandgap, where the HH orders was continuously controlled by the carrier-envelop phase of the THz pulse. The non-integer multiples of the HH generation occur when the Dirac electrons traverse Dirac point ballistically, allowing to discern between the TSS dynamics and the bulk response.

## Hybrid systems of graphene and 3D topological insulator

**Fig. 5:**
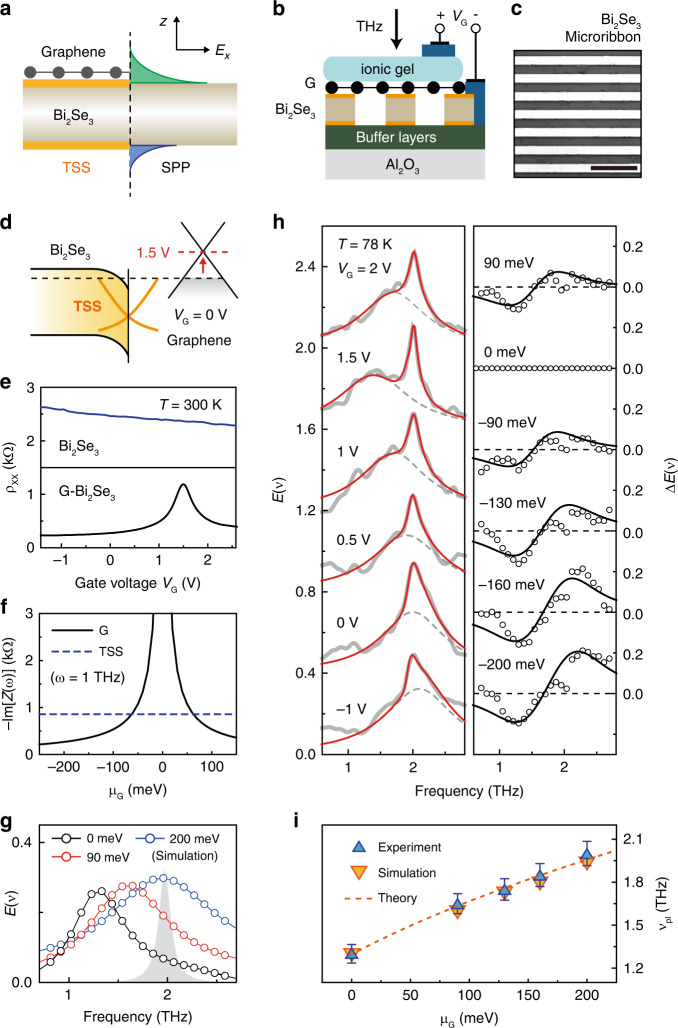
Electric control of graphene and 3D TI plasmon in a hybrid structure. **a** Graphene is transferred on top of Bi_2_Se_3_ topological surface state (TSS). The electric field *E*_*x*_ profiles of the surface plasmon-polariton (SPP) decay along with the *z*-direction. **b** Terahertz (THz) is incident on graphene (G) and Bi_2_Se_3_ microribbon (G-Bi_2_Se_3_), where the Bi_2_Se_3_ is grown on (Bi_0.5_In_0.5_)_2_Se_3_/Al_2_O_3_ substrate. The gate voltage V_G_ is applied to the transparent ionic gel. **c** Optical microscope image of the 20-μm-width Bi_2_Se_3_ microribbon. The scale bar corresponds to 100 μm. **d** The bandstructures of the Bi_2_Se_3_ and graphene interface are displayed, where the surface band-bending in Bi_2_Se_3_ arises from charged impurities. The TSS (graphene) is in an *n*-doped (*p*-doped) state in equilibrium. The V_G_ primarily changes the graphene chemical potential. **e** Longitudinal resistances *ρ*_*xx*_ at 300 K are measured as a function of V_G_ in Bi_2_Se_3_ and G-Bi_2_Se_3_. **f** Imaginary part of impedances *Z*(*ω*) (*ω* = 1 THz) in G and TSS are calculated at different values of the graphene chemical potential *μ*_G_. **g** Spectral extinction *E*(*ν*) in G-Bi_2_Se_3_ is simulated at *μ*_G_ = 0, 90, and 200 meV. The gray area indicates the Bi_2_Se_3_ phonon resonance. **h** Left: experimental *E*(*ν*) (solid gray line) at 78 K and fit to the data (red line) are displayed at different V_G_. The bare plasmon extinction (gray dashed line) is extracted from the fit line. Right: extinction differences Δ*E*(*ν*) = *E*(*ν*)−*E*_0_(*ν*) (circles) are displayed, where *E*_0_(*ν*) corresponds to the extinction at *V*_G_ = 1.5 V (*μ*_G_ = 0 meV). Black lines are obtained from the difference in the bare plasmon extinction. **i** Experimental (blue triangles) and simulated (orange triangles) plasmon frequency *ν*_pl_. Fit line follows the optical plasmon mode in G-Bi_2_Se_3_ (orange dashed line)

Next, we explore the electrostatic modulation of the TSS plasmon integrated with graphene. Unlike the case of graphene, the large dielectric constant of Bi_2_Se_3_ at low frequency (*κ*_TI_ = 100) prevents the electrostatic doping, effectively screening the gate-induced electric field. Previously, thin layers of Bi_2_Se_3_ (~10 nm) and molecular charge transfers are used to demonstrate the ambipolar field-effect of the TSS^87,88^, otherwise the gate-voltage *V*_G_ barely charges the Bi_2_Se_3_ carrier density. Nonetheless, one can electrically control the TSS plasmon by integrating a monolayer CVD-grown graphene on top of Bi_2_Se_3_ TI microribbons, where the *V*_G_ can tune the graphene chemical potential *μ*_G_, changing the dielectric environment of the surrounding Bi_2_Se_3_. Indeed, the dynamic screening effect of monolayer graphene modulates the SPP response in Bi_2_Se_3_ (Fig. 5a). Figure 5b–e illustrate the graphene and 30-QL Bi_2_Se_3_ microribbon (G-Bi_2_Se_3_) device and the field-effect, where the transparent ionic-gate controls *μ*_G_. While the longitudinal resistance *ρ*_*xx*_ of Bi_2_Se_3_ remains at ~2.5 kΩ, the ambipolar field-effect on G-Bi_2_Se_3_ illuminates that the monolayer graphene is in a p-doped state before applying *V*_G_. To appreciate the SPP response in a doped G-Bi_2_Se_3_, we performed simulations using the 3D-EM simulator (High-Frequency Structure Simulator), where the impedance boundary conditions *Z*(*ω*) = 1/*σ*(*ω*) incorporate the Drude sheet conductance *σ*(*ω*) = *D*/(Γ − i*ω*). Here, the Drude weight *D* = (*e*^2^/4*π*ℏ^2^)*g*_*s*_*g*_*v*_*μ*_TSS/G_ and the scattering rate Γ determine the density-dependent reactance −Im[*Z*(*ω*)] (Fig. 5f) with the chemical potentials of TSS/graphene (*μ*_TSS/G_).

Using *μ*_TSS_ of 250 meV and the gate-dependent *μ*_G_ ranging from 0 meV to 200 meV, we can reproduce the density-dependent wave extinction *E*(*ν*) = 1 − *t*/*t*_0_ spectrum of the G-Bi_2_Se_3_ (Fig. 5g), where the plasmon frequency *ν*_pl_ undergoes a blue-shift when *μ*_G_ increases. Here, the Bi_2_Se_3_ phonon resonance at ~2 THz is excluded from the simulation, which still serves as a reliable tool to track the density-dependent SPP response in the G-Bi_2_Se_3_. Indeed, the THz measurements show that the plasmonic response of G-Bi_2_Se_3_ changes at different *V*_G_ (Fig. 5h). We plot the extinction differences Δ*E*(*ν*) relative to the undoped G-Bi_2_Se_3_ (*V*_G_ = 1.5 V) to isolate the plasmonic resonance from the phonon resonance at ~ 2 THz, where the spectral blue-shift becomes evident at the higher carrier density. The bare plasmon extinction (gray dashed line) can also be extracted from the fit function (red line). The experimental *ν*_pl_ shows an excellent agreement with the simulated *ν*_pl_ when we assume *μ*_G_ = 200 meV at *V*_G_ = −1 V, which corresponds to the gate capacitance ~1.2 × 10^12^ cm^−2^. Figure 5i highlights the observation, where the experimental and simulated *ν*_pl_ values are almost identical at all doped status of G-Bi_2_Se_3_. One intriguing aspect of G-Bi_2_Se_3_ is that the density-dependence in *ν*_pl_ is different from the standard 2D Dirac plasmon $\nu _{{{{\mathrm{pl}}}}}^2 \propto g_sg_v\mu _{{{\mathrm{G}}}}$. Recalling that the *μ*_TSS_ is independent of *V*_G_ in the device, the density-dependence in G-Bi_2_Se_3_ is written as $\nu _{{{{\mathrm{pl}}}}}^2 \propto 2\mu _{{{{\mathrm{TSS}}}}} + 4\mu _{{{\mathrm{G}}}}$, where the pre-factor 2 at *μ*_TSS_ arises from the top and bottom TSS. For this reason, the dynamic polarization of graphene on top of Bi_2_Se_3_ can efficiently control the SPP response, achieving the *ν*_pl_ modulation ~50% within the *V*_G_ range.

## Applications and future outlooks

**Fig. 6:**
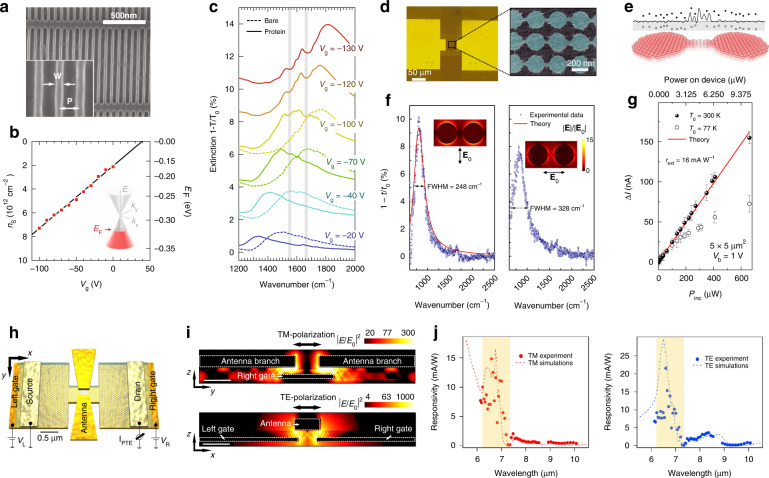
Optoelectronic applications using graphene plasmon-polariton. **a** Scanning electron microscope image of graphene nanoribbon (*W* = 30 nm and *P* = 80 nm). **b** Graphene carrier density *n*_*s*_ and Fermi energy *E*_F_ are shown as a function of the gate voltage *V*_*g*_. **c** Infrared extinctions are measured for bare graphene nanoribbon (dashed lines) and with protein formation (solid lines) at different *V*_*g*_. Vertical gray lines indicate the vibrational modes of protein. **d** Left: optical microscope image of the device. Right: scanning electron micrograph image of the graphene nanodisks. **e** Schematic illustration of disorder potentials in graphene nanoribbon connecting the graphene nanodisks. Laser excitation creates thermal distribution of electron (black dots) and hole (white dots) over the disorder potential. **f** Spectral extinctions (dots) are measured with electric field **E**_**0**_ perpendicular (left) and parallel (right) to the graphene nanoribbon. The full-width half-maximum (FWHM) is obtained from the theoretical fit to the data (red line). Inset shows the electric field distribution at the resonance. **g** The pump-induced changes in the electric current ∆*I* at 77 K (black dots) and 300 K (white dots) are plotted as a function of incident power *P*_inc_. The external responsivity *r*_ext_ is obtained at the device area 5 × 5 μm^2^ with the source-drain bias voltage *V*_b_ = 1 V. The theoretical fit line explains the *P*_inc_-dependent ∆*I*. **h** Monolayer graphene encapsulated with hBN is transferred onto resonant H-shape gates. The bow-tie antenna on top of the device is resonantly coupled with infrared radiation. **i** Top: pump electric field is resonantly coupled with the bow-tie antenna (TM polarization). Bottom: electric field excites a resonance at the H-shape gate (TE polarization). The near-field coupling launches HPP mode inside the hBN layer. **j** Experimental (dots) and simulated responsivity (dashed lines) are shown with TM (left) and TE polarizations (right). The shaded region indicates the HPP band inside hBN. **a‒c** Adapted with permission from ref. ^89^, American Association for the Advancement of Science. **d**‒**g** Adapted with permission from ref. ^51^, Springer Nature. **h**‒**j** Adapted with permission from ref. ^95^, Springer Nature

The extraordinary near-field interaction of graphene plasmon and molecular vibrations can be used to develop highly efficient molecular sensors detecting proteins^89^, gas molecules^90^, and polar phonons^46^. Figure 6a–c highlight the molecular detection of mid-IR graphene plasmon, where the gate-tuned graphene nanoribbon serves as an efficient tool to detect the vibrational bands of protein. Upon the protein immobilization, the screening effect induces the red-shift in the plasmon resonance (Fig. 6c), where the ripples at 1550 cm^−1^ and 1660 cm^−1^ provide the vibrational fingerprints of the protein. The sensitive molecular detection of the graphene plasmon is ascribed to the strong confinement of near-field distribution up to a few nanometers from the surface. In contrast, the near-field distribution of gold plasmons reaches hundreds of nanometers at the same resonance frequency, resulting in a lower sensitivity to the vibrations of the molecular band.

The mid-IR responsivity of graphene plasmon can be boosted by stacking multi-layers of graphene. Guo et al.^51^ demonstrated a mid-IR photodetector based on the graphene nanodisk stacks (Fig. 6d–g), where the photoinduced carriers are transported through the graphene nanoribbons connecting the graphene nanodisks. Before the incidence of mid-IR photons, the disorder potentials of graphene nanoribbon localize electron wavefunctions, functioning as a bottleneck to the electric current. Upon the mid-IR incidence, a sharp resonance occurs at the graphene disk (Fig. 6f), increasing the temperature of the localized states. Schematics in Fig. 6e illustrate that the thermal excitation can delocalize the carriers away from the disorder potential, activating the current channel of the mid-IR photodetector. The temperature-dependent photoresponse in Fig. 6g indicates that the thermal carrier excitation is responsible for the photocurrent generation, achieving an external responsivity of 16 mA W^−1^ at room temperature.

A range of graphene-based optoelectronic applications utilizes unique graphene photoresponses, integrating graphene with waveguides^91^, metallic surface plasmons^92^, and plasmonic nanostructures^93,94^. Castilla et al.^95^ reported that the hyperbolic phonon-polaritons (HPP) of hBN enhance the mid-IR responsivity of the graphene field-effect device. Figures 6h–j show that the graphene photodetector is combined with a plasmonic antenna, where metallic dual-gates and bow-tie antenna are designed to show resonances at the HPP band. As displayed in Fig. 6i, the constructive interference between the HPP mode and the plasmonic resonance amplifies the light absorption. As a result, the prominent enhancement in the near-field interaction at the active area drives a non-equilibrium thermal distribution of the graphene field-effect device, converting the mid-IR photon energy into the thermoelectric photocurrent. The responsivity of the device exhibits a resonant responsivity ~15 mA W^−1^ inside the HPP band at both TM and TE pump polarizations (Fig. 6j).

From the viewpoint of spintronics, the 3D TI helical spin texture provides promising tools to investigate the spin-charge coupled phenomena^96–98^: the longitudinal charge current at the TI surface can induce the transverse spin polarization and vice versa. The spin texture of 3D TI is analogous to the Rashba state observed in 2D electron gas (2DEG) but intrinsically distinguished since the spin texture itself constructs the surface state in the 3D TI. The spin conversion efficiency in the TI surface is superior to the conventional Rashba state in 2DEG because the TI Fermi surface consists of a single helical spin texture. In contrast, the Fermi energy in 2DEG intersects with co-centric double Fermi contours with opposite spin helicity. Meanwhile, the light-matter interaction brings significant aspects to the Dirac surface states. The obliquely-incident photon helicity can be coupled with the in-plane spin texture of the 3D TI surface^99^, where the corresponding spin depletion leads to the transverse charge current. As a result, the helicity-dependent photocurrent captures the unique helical spin texture of the 3D TI surface. In a ferromagnet and 3D TI bilayer, light illumination on the ferromagnetic layer injects spin into the 3D TI surface^100^. The spin is efficiently converted into charge current via the inverse Edelstein effect, where the surface charge current manifests itself in the broadband THz radiation.

Versatile optoelectronic applications of 2D Dirac plasmon stem from the highly confined near-field distribution on the surface at the wide range of IR wavelength. Employing the non-local 2D Dirac polarization, we have shown that the plasmon dispersion undergoes remarkable modifications under the plasmon-phonon coupling, plasmon-plasmon coupling, and interband thermal excitations. Both far- and near-field detections of 2D Dirac plasmon have been performed at IR wavelength, allowing us to characterize the comprehensive plasmonic response for device applications. For instance, the thermoelectric current in a novel dual-gate device could detect the acoustic mode of graphene plasmon-polariton, confining the plasmon wavelength into the deep-subwavelength scale. Meanwhile, intense optical and THz pulses can trigger non-equilibrium thermal distribution at the 2D Dirac-band, changing the plasmon dispersion of graphene and 3D TI Bi_2_Se_3_. Integrating the graphene and Bi_2_Se_3_, we could control the Bi_2_Se_3_ plasmon frequency by changing the graphene Fermi energy, revealing the extraordinary response of near-field interactions in close proximity. The outstanding properties of 2D Dirac plasmon can be employed to develop future mid-IR photodetectors, bio-molecular sensors, THz detectors, and light sources.
